# Molecular and Phenotypic Characterization of Multidrug-Resistant *Aspergillus fumigatus* Clinical Isolates in Republic of Korea

**DOI:** 10.3390/jof12050302

**Published:** 2026-04-22

**Authors:** Yun Ha Lee, Yewon An, Yu Jin Lee, Jihee Lee, Su Yeon Kim, Byung Hak Kang

**Affiliations:** 1 Division of Pathogen Resource Management, Center for Vaccine Research, National Institute of Health, Korea Disease Control and Prevention Agency (KDCA), Cheongju 28160, Republic of Korea; yuna9092@korea.kr (Y.H.L.);; 2 Division of Bacterial Disease, Bureau of Infectious Disease Diagnosis Control, Korea Disease Control and Prevention Agency (KDCA), Cheongju 28160, Republic of Korea

**Keywords:** *Aspergillus fumigatus*, antifungal resistance, genetic diversity, *cyp51A*, *HMG1*, *FKS1*, whole-genome sequencing

## Abstract

Genetic diversity and antifungal susceptibility profiles of *Aspergillus fumigatus* are critical for understanding the evolution of resistance in clinical and environmental settings. We performed comprehensive genomic characterization of *A. fumigatus* isolates using whole-genome sequencing combined with phenotypic susceptibility assays. SnpEff-based variant annotation identified 76,079 single-nucleotide polymorphisms, revealing a high proportion of mutations (78.8%) in upstream and downstream regulatory regions, whereas high-impact coding variants remained rare (0.083%). Several key mutations were identified, including the well-established *cyp51A* M220V and *HMG1* S212P/Y564H mutations. Moreover, a diverse array of peripheral *cyp51A* polymorphisms (M39I, E402D, N248K, and K372N) was detected, although these variants did not correlate with the resistant phenotypes. Our comparative genomic analysis identified a novel A586T substitution in the *FKS1* gene in an isolate with an elevated minimum effective concentration of caspofungin, suggesting its possible association with reduced susceptibility, although functional validation is required. In isolates lacking canonical target-site mutations, the high frequency of regulatory-region variants indicated the involvement of non–target-site mechanisms. This study provides a detailed map of the genomic landscape of *A. fumigatus* and identifies candidate loci for future functional validation. Our results demonstrate the utility of high-throughput genomic surveillance for monitoring emerging resistance trends and characterizing the genetic background of clinical fungal pathogens.

## 1. Introduction

*Aspergillus fumigatus* is a ubiquitous saprophytic fungus and the primary cause of invasive aspergillosis (IA), a life-threatening condition with high morbidity and mortality in immunocompromised individuals. In recognition of the escalating threat posed by fungal infections, the World Health Organization released its first Fungal Priority Pathogens List in 2022, categorizing *A. fumigatus* within the “Critical Priority” group [1]. This designation underscores the urgent need for global surveillance and the development of new therapeutic strategies.

Azole agents, such as voriconazole, are currently the first-line gold standard for the treatment of IA, as recommended by the Infectious Diseases Society of America and European Society for Clinical Microbiology and Infectious Diseases [2]. As a landmark study by Herbrecht et al. [3] demonstrated the superior efficacy of voriconazole over amphotericin B (AMB), triazoles have been the cornerstone of antifungal therapy. Consequently, the emergence of azole-resistant *A. fumigatus* poses a major clinical concern because it severely limits therapeutic options and is associated with significantly increased mortality rates [4,5,6].

Resistance development in *A. fumigatus* is driven by two distinct selective pressures. The clinical route occurs during long-term antifungal treatment in individual patients, leading to diverse point mutations in the *cyp51A* gene [7]. In contrast, the environmental route is associated with the extensive use of azole fungicides in agriculture, which have molecular structures similar to those of medicinal triazoles. This environmental selection led to the widespread prevalence of specific resistance markers, notably TR34/L98H and TR46/Y121F/T289A, which are often found in azole-naïve patients.

In Republic of Korea, the prevalence of azole-resistant *A. fumigatus* has historically been considered lower than that in European countries. However, recent surveillance data have indicated the steady emergence of resistant clinical isolates [8]. Notably, the TR34/L98H mutation in the cyp51Agene, a well-recognized environmental resistance mechanism, has been increasingly reported worldwide, highlighting the global spread of azole resistance. In line with this trend, such environmental resistance markers have also been identified in Brazil, raising concerns about the shifting epidemiology of fungal infections [9,10]. Despite these reports, longitudinal studies characterizing long-term trends in antifungal susceptibility remain scarce.

Therefore, this study aimed to characterize the antifungal susceptibility profiles and molecular mechanisms of resistance in *A. fumigatus* clinical isolates collected by the National Culture Collection for Pathogens (NCCP) in Republic of Korea between 2012 and 2022. By integrating phenotypic susceptibility testing with molecular and genomic analyses, this study provides important insights into the evolving landscape of resistance and contributes valuable data for regional surveillance and clinical management of aspergillosis.

## 2. Materials and Methods

### 2.1. Strains

Sixteen *A. fumigatus* strains were obtained from the NCCP of the Republic of Korea Disease Control and Prevention Agency. These human clinical isolates were collected over a 10-year period (2012–2022) from various healthcare institutions across Republic of Korea, including the provinces of Gangwon, Daejeon, and Jeollanam (Table S1).

### 2.2. DNA Extraction

Genomic DNA was extracted from the inactivated *A. fumigatus* mycelia using a modified cetyltrimethylammonium bromide (CTAB)/phenol:chloroform:isoamyl alcohol (PCI) method. Briefly, 100 mg of mycelia was pulverized with 5-mm stainless steel beads using a Qiagen TissueLyser. The disrupted mycelia were resuspended in 1 mL of specialized lysis buffer (containing Buffer A, B, C, and PVP40, and PVP10) and incubated at 65 °C for 30 min, followed by protein digestion with Proteinase K (20 mg/mL) (Thermo Fisher Scientific, USA). The proteins and polysaccharides were precipitated on ice using 5 M potassium acetate. The supernatant was subjected to double purification with PCI 25:24:1 Saturated with 10 mM Tris, pH 8.0, 1 mM EDTA (Sigma-Aldrich, Germany). After RNase A (Thermo Fisher Scientific, USA) treatment (10 mg/mL) to remove RNA, the DNA was precipitated with 3 M sodium acetate and isopropanol. The resulting DNA pellet was washed twice with 70% ethanol, air-dried, and finally dissolved in 100 μL of TE buffer or Tris-HCI buffer [11]. DNA concentration was measured using a NanoDrop spectrophotometer, and DNA integrity was confirmed by electrophoresis on a 0.7% agarose gel.

### 2.3. Antifungal Susceptibility Profiles (Minimum Inhibitory Concentration Values)

The minimum inhibitory concentrations (MICs) of the azoles (fluconazole, itraconazole, voriconazole, and posaconazole), Flucytosine, and amphotericin B, and minimum effective concentrations (MECs) echinocandins (caspofungin, micafungin, and anidulafungin), and AMB were determined using the Sensititre YeastOne panel (Thermo Fisher Scientific, USA), according to the manufacturer’s instructions which follow the CLSI M38 guidelines. Susceptibility categorization was interpreted according to the CLSI breakpoints or epidemiological cutoff values, as appropriate. MIC values are presented in µg/mL.

### 2.4. Mutations Identified in Antifungal Resistance-Associated Genes

To identify mutations associated with antifungal resistance, hotspot regions of *cyp51A*, *HMG1*, and *FKS1* were amplified using polymerase chain reaction (PCR). The primers were designed based on the reference sequence of *A. fumigatus* Af293 (GenBank accession no. NC_007194.1). Primer sequences and their respective annealing temperatures are summarized in Table S2. PCR amplification was performed using a Phusion™ High-Fidelity DNA polymerase (NEB, USA), following the PCR conditions described in Table S3 [12,13,14]. The resulting amplicons were purified using a AccuPrep^®^ PCR/Gel Purification Kit (Bioneer, Republic of Korea) and sequenced using Sanger sequencing.

### 2.5. Whole-Genome Sequencing

Among the 16 isolates, three representative strains (CF3349, 22455, and 32662) were selected for whole-genome sequencing (WGS) based on their distinct multidrug-resistant or high-MIC phenotypes to further investigate the underlying genomic mechanisms. Genomic DNA from strains 22455, 32662, and CF3349 was extracted using the CTAB method. Quality control analysis indicated DNA concentrations of 16.55, 13.08, and 14.24 nM, all meeting the requirements for sequencing. The total number of bases, reads, GC (%), and Q30 (%) were calculated for each sample. Whole-genome sequencing (WGS) was performed using an Illumina platform with paired-end reads (2 × 151 bp). A total of approximately 22.3 million reads were generated, resulting in an average genome coverage of approximately 200×. The high-quality reads were trimmed and mapped to the reference genome of *A. fumigatus* AF293 (GCF_000002655.1). The assembly was conducted using Hifiasm version 0.19.9, and short-read polishing was performed using Pilon version 1.22. The Whole Genome Sequencing datasets for this study can be found in the NCBI BioProject repository (PRJNA1436041) via the following BioSample accessions: SAMN56459848 (22455), SAMN56459849 (32662), and SAMN56459850 (CF3349).

Functional annotation of the identified variants was performed using SnpEff (version 5.1). A custom database for *A. fumigatus* was built using SnpEff based on the reference GTF/GFF3 annotation files. Variants were categorized by predicted biological impact as high (nonsense mutations and frameshifts), moderate (missense mutations), low (synonymous mutations), and modifier (e.g., upstream, downstream, and intergenic variants).

## 3. Results

### 3.1. Antifungal Susceptibility Profiles

Although clinical breakpoints for echinocandins in *Aspergillus fumigatus* have not been established by the CLSI **or** EUCAST, 14 strains, including 21549, 21550, 22455, and 32662, exhibited high MICs of caspofungin (>8 µg/mL). Among these isolates, several showed high-level echinocandin resistance, with MICs of ≥4 µg/mL for anidulafungin and >8 µg/mL for micafungin. Additionally, 13 strains, excluding 22482, 32663, and MFCSSHM18224, demonstrated MICs ≥ 32 µg/mL for flucytosine. Most isolates had AMB MICs ranging from 1 to 3 µg/mL, falling within the resistant (R) category. However, strains CF3110 and MFCSSHM18224 were identified as susceptible (S) based on the established breakpoints. Regarding azole antifungals, most isolates were susceptible to itraconazole and voriconazole but exhibited high MICs (≥64 µg/mL) for fluconazole. CF3349 displayed a broad antifungal resistance profile, showing resistance to AMB (2 µg/mL) and echinocandins (>8 µg/mL) and triazoles, including posaconazole (2 µg/mL) and voriconazole (>8 µg/mL), with the exception of itraconazole (1 µg/mL) (Table 1).

### 3.2. Mutations Identified in Antifungal Resistance-Associated Genes

To comprehensively profile the genetic variants of this strain, we used a combination of WGS and targeted sequencing of specific antifungal resistance-related genes. In 13 of the 16 analyzed strains, S212P and Y564H mutations in the *HMG1* gene were concurrently identified. These mutations were observed as common features in both susceptible and resistant strains. Regarding *cyp51A*, a major target for azole resistance, several amino acid substitutions were confirmed, including K372N (21550), M220V (MFCSSHM18224), N248K (MFCUSHM00023), and M39I/E402D (MFCDEHM00006). Conversely, no insertion mutations in the *cyp51A* promoter region (e.g., TR34 and TR46) were detected in any isolate. In the *FKS1* gene, which is associated with echinocandin resistance, the A586T (32663) and G1199S (CF3350) mutations were identified in a single strain.

Using WGS analysis, we identified specific variants within the ergosterol biosynthesis pathway of strain 22455. Notably, two missense mutations, V196M and C628R, were confirmed in the *HMG1* gene, which is a known hotspot for antifungal resistance-related alterations. In contrast to strain 22455, WGS and targeted sequencing of strains 32662 and CF3349 revealed only the C628R substitution without the concomitant presence of the V196M mutation (Table 2).

### 3.3. Whole Genome Sequencing and General Genomic Variation

Based on their distinct antifungal susceptibility profiles, three isolates (22455, 32662, and CF3349) exhibiting the highest MIC values were selected for WGS analysis. Strains 22455 and 32662 were prioritized owing to their notably high MICs to AMB, whereas CF3349 was selected for its multidrug-resistant phenotype across several antifungal classes.

After filtering, the number of identified single-nucleotide polymorphisms (SNPs) ranged from 69,860 to 86,134, corresponding to an average variant rate of one SNP per 341–420 bp relative to the reference genome (29.4 Mb) (Table 3 and Table 4). In all three isolates, most variant effects were classified as “modifier” variants (~96.0%), whereas “high-impact” variants that could significantly alter protein function remained significantly low (0.05–0.08%). Regarding the functional classification of coding variants, missense mutations accounted for the largest proportion (51.6–51.9%), followed by silent mutations (45.5–47.1%). Positional analysis revealed that approximately 79% of the variants were located in the upstream and downstream regions, indicating that most genetic variations occurred outside gene coding regions.

## 4. Discussion

The mechanisms of azole resistance in *A. fumigatus* are primarily associated with modifications in the sterol biosynthesis pathway, particularly through amino acid substitutions in the *cyp51A* protein or insertion of tandem repeats in its promoter region. Although TR34/L98H remains the dominant genotype globally, recent studies have reported that S212P and Y564H mutations also significantly contribute to azole resistance [15]. In the present study, we identified a diverse range of mutations in the *cyp51A* gene, including M220V, M39I, E402D, and N248K. Among these, M220V is a well-established resistance-conferring mutation located near the channel opening of the *cyp51A* protein, where it physically obstructs the entry of long-side-chain azoles. In contrast, based on structural homology models, mutations such as K372N, M39I, E402D, and N248K are located at the protein periphery, far from the heme-active site. These alterations were predicted to have negligible effects on azole-binding affinity or structural integrity, functioning as neutral genetic polymorphisms rather than resistance determinants [16].

The presence of these peripheral *cyp51A* mutations in our isolates underscores the complexity of genotypic interpretation, as they do not consistently correlate with a resistant phenotype. In line with this, recent work by Song et al. [17] also demonstrated that variation in *cyp51A* does not always directly correlate with azole susceptibility, highlighting genotype–phenotype discordance. Consistent with these findings, our results further support the notion that cyp51A variation alone may be insufficient to explain antifungal resistance. This observation is also consistent with our analysis of the *HMG1* gene, in which the S212P and Y564H mutations were identified. Unlike the neutral polymorphisms in *cyp51A*, these *HMG1* substitutions, particularly those residing in the sterol-sensing domain, may contribute to resistance; however, their functional significance remains uncertain without experimental validation. Consequently, our findings emphasize that a comprehensive resistance profile cannot be achieved by *cyp51A* sequencing alone; rather, it requires the simultaneous evaluation of *HMG1* to distinguish functional resistance mutations from incidental genetic backgrounds.

Echinocandin resistance is typically associated with mutations within two highly conserved regions of the *FKS1* protein, known as hotspot 1 (e.g., S673 and F675) and hotspot 2 (e.g., P1305) [14,18]. In our study, we identified two non-synonymous mutations outside of these conventional hotspots: A586T and G1199S. Although these substitutions do not reside within the canonical resistance-determining regions, their clinical significance appears to be distinct based on our phenotypic data. Notably, isolate 32663, harboring the A586T mutation, exhibited a significantly elevated minimum effective concentration (MEC) for caspofungin. This high MEC value suggests that A586T may be associated with reduced susceptibility, representing a potential candidate mutation; however, its functional role requires further validation. Conversely, the G1199S mutation found in isolate CF3350 was associated with a low MEC, similar to that in the wild-type strains. This phenotypic susceptibility led us to interpret G1199S as a neutral genetic variant or simple polymorphism, rather than as a functional determinant of resistance. These findings highlight the importance of integrating phenotypic susceptibility testing with genotypic analysis to identify emerging resistance loci beyond well-characterized hotspots of the *FKS1* gene.

The absence of characterized mutations in well-known resistance loci, specifically *cyp51A* (azoles) and *FKS1* (echinocandins), is particularly significant. Despite the absence of these canonical markers, the observed variations in fungicide susceptibility suggest that resistance in this strain may be mediated by non–target-site mechanisms. Such phenotypes can arise from overexpression of efflux transporters, such as *CDR1B* [19], or from alterations in transcriptional regulators, such as *AtrR* [20], which modulate the expression of multiple resistance-related genes without altering the target sequence itself.

In addition to target-site mutations, our WGS analysis identified multiple variants in genes encoding MFS and ABC transporters. These transporters are known to contribute to antifungal resistance through drug efflux mechanisms, potentially reducing intracellular drug accumulation and thereby decreasing susceptibility. Furthermore, variants were also detected in cytochrome P450-related genes, which may influence sterol metabolism or drug processing. Although the direct contribution of these mutations to resistance remains unclear, their presence in our dataset supports the involvement of non–target-site mechanisms. Taken together, these findings suggest that antifungal resistance in these isolates may be multifactorial and not solely dependent on canonical target-site mutations.

In line with this, regulatory mechanisms may also play an important role in modulating antifungal susceptibility. Deletion of *SrbA*, a direct transcriptional regulator of *cyp51A*, significantly reduces *cyp51A* expression, resulting in a hypersusceptible phenotype [21]. These findings suggest that mutations in transcriptional regulators or variations in their expression levels can critically influence antifungal resistance mechanisms. Furthermore, given the high frequency of upstream variants identified in our genomic analysis, it is plausible that these mutations affect the binding affinity of regulators, such as *SrbA*, thereby contributing to the observed variations in fungicide susceptibility despite the absence of canonical coding mutations.

Although the V196M mutation in *HMG1* has been previously reported as an azole resistance marker [22], our isolate remained susceptible to triazoles. This suggests that the V196M variant alone may be insufficient to confer high-level resistance and may require an additional genetic background or environmental selective pressure to fully manifest a resistant phenotype. In addition, the C628R substitution is a variant located in a relatively less-characterized region of the *HMG1* gene. Given its rarity, further functional studies, such as gene replacement or protein structural analyses, are warranted to elucidate its specific impact on antifungal susceptibility and ergosterol biosynthesis.

The high proportion of missense mutations (>51%) and the presence of hundreds of high-impact variants highlighted the significant genetic diversity among the isolates, suggesting their potential contribution to antifungal resistance phenotypes. Specifically, 8778 missense mutations identified in strain 22,455 included the aforementioned *HMG1* variants. These genetic alterations may underlie atypical drug responses, such as AMB resistance, even in the absence of triazole resistance.

This study has several limitations. First, detailed clinical information, including antifungal exposure history, was not available, limiting our ability to distinguish between clinically acquired and environmentally derived resistance. Second, although mutations in genes such as HMG1 were identified, their functional significance remains unclear without experimental validation. Third, no clear association was observed between specific mutations and MIC patterns, suggesting that antifungal resistance may be multifactorial. In addition, structural analyses of the identified mutations were not performed, which limits our ability to interpret their mechanistic impact. Finally, the relatively small sample size (n = 16) limits the generalizability of our findings, and further studies with larger datasets are required.

## 5. Conclusions

Azole and echinocandin resistance in *A. fumigatus* is a multifaceted phenomenon that extends beyond well-known target-site mutations. The identification of novel candidate loci, along with the high prevalence of regulatory-region variants, underscores the necessity of a comprehensive pan-genomic approach. Furthermore, studying the genetic diversity and antifungal susceptibility profiles of *A. fumigatus* has important implications in both clinical and environmental settings: Clinically, it contributes to the optimization of antifungal therapy and effective antifungal stewardship. Environmentally, it facilitates the surveillance of resistant strains and helps elucidate transmission dynamics between environmental and clinical sources, thereby contributing to improved public health strategies.

## Figures and Tables

**Table 1:** Antifungal susceptibility profiles (MIC values) of 16 *Aspergillus fumigatus* isolates.

Strain (NCCP) No.	AMB		ANF	CAS	FLC	5-FC	ITC		MCF	POS		VOR	
	MIC	R/S	MIC	MIC	MIC	MIC	MIC	R/S	MIC	MIC	R/S	MIC	R/S
21549	2	R	>8	>8	>256	64	0.25	S	>8	0.12	S	0.5	S
21550	2	R	>8	>8	>256	64	0.25	S	>8	0.12	S	0.25	S
22455	2.7	R	4	>8	>256	>64	0.5	S	>8	0.25	S	0.5	S
22482	1.67	R	4	>8	>256	8	0.25	S	>8	0.12	S	0.33	S
32662	4	R	4	>8	>256	64	0.25	S	>8	0.25	S	0.25	S
32663	2	R	8	>8	>256	0.25	0.06	S	>8	0.03	S	0.25	S
CF3289	2	R	8	>8	>256	>64	0.25	S	>8	0.06	S	0.25	S
CF3290	2	R	4	>8	>256	>64	0.25	S	>8	0.06	S	0.5	S
CF3348	2	R	4	>8	>256	>64	0.12	S	>8	0.06	S	0.25	S
CF3349	2	R	>8	>8	>256	>64	1	S	>8	2	R	>8	R
CF3350	2	R	0.015	0.03	>256	>64	0.25	S	0.008	0.06	S	0.25	S
MFCJUHM00002	2	R	4	>8	>256	32	0.12	S	>8	0.06	S	0.25	S
MFCUSHM00023	2.7	R	4	>8	>256	>64	0.12	S	>8	0.06	S	0.25	S
MFCSSHM18224	0.12	S	0.015	0.03	>256	1	0.29	S	0.008	0.21	S	0.14	S
MFCDEHM00006	2	R	4	>8	>256	>64	0.25	S	>8	0.12	S	0.5	S
MFCUSHM00065	2	R	4	>8	>256	>64	0.5	S	>8	0.12	S	0.5	S

Minimum inhibitory concentrations (MICs) for azoles (fluconazole, itraconazole, voriconazole, posaconazole), echinocandins (caspofungin, micafungin, anidulafungin), and amphotericin B were determined using the CLSI broth microdilution method. Susceptibility categorization was interpreted according to CLSI breakpoints or epidemiological cutoff values (ECVs), as appropriate. Notably, only itraconazole resistance was observed among the isolates. MIC values are presented in µg/mL. (Abbreviations: AMB, amphotericin B; ANF, anidulafungin; CAS, caspofungin; FLC, fluconazole; 5-FC, flucytosine; ITC, itraconazole; MCF, micafungin; POS, posaconazole; VOR, voriconazole).

**Table 2:** Mutations identified in antifungal resistance-associated genes among seven selected *Aspergillus fumigatus* isolates by targeted sequencing or WGS.

Strain (NCCP) No.	*HMG1*	*cyp51A*	*FKS1*	Remarks
21549	S212P, Y564H	-	-	Targeted sequencing
21550	S212P, Y564H	K372N	-	Targeted sequencing
22455	V196M, C628R	-	-	WGS
22482	S212P, Y564H	-	-	Targeted sequencing
32662	C628R	-	-	WGS
32663	S212P, Y564H	-	A586T	Targeted sequencing
CF3289	S212P, Y564H	-	-	Targeted sequencing
CF3290	S212P, Y564H	-	-	Targeted sequencing
CF3348	S212P, Y564H	-	-	Targeted sequencing
CF3349	C628R	-	-	WGS
CF3350	S212P, Y564H	-	G1199S	Targeted sequencing
MFCDEHM00006	S212P, Y564H	M39I, E402D	-	Targeted sequencing
MFCJUHM00002	S212P, Y564H	-	-	Targeted sequencing
MFCSSHM18224	S511C, Y564H	M220V	-	Targeted sequencing
MFCUSHM00023	S212P, Y564H	N248K	-	Targeted sequencing
MFCUSHM00065	S212P, Y564H	-	-	Targeted sequencing

This table summarizes amino acid substitutions detected in *HMG1*, *cyp51A,* and *FKS1*. For each isolate, the gene, position, and predicted amino-acid subsititution are reported. Dash (-) indicates no mutation or insertion.

**Table 3:** Summary of WGS statistics and quality metrics for three *Aspergillus fumigatus* isolates.

Strain (NCCP) No.	Assembly Method	Total Length	Total Number of Bases	Q30	GC Content (%)
**22455**	Illumina	11,218,724	3,388,054,648	95.52	48.38
**32662**	Illumina	9,938,109	3,001,308,918	95.41	48.71
**CF3349**	Illumina	11,459,629	3,460,807,958	95.41	48.48

**Table 4:** Statistics of single-nucleotide polymorphisms (SNPs) and classification of variants based on their functional impact in each isolate.

Parameters	22455	32662	CF3349
**Total SNPs (filtered)**	69,860	76,079	86,134
**Variant rate (1 per bp)**	420 bp	386 bp	341 bp
**Functional class: missense (count)**	8778 (51.66%)	9639 (51.653%)	11,228 (51.895%)
**Functional class: nonsense (count)**	218 (1.283%)	333 (1.784%)	202 (0.934%)
**High impact variants (count)**	301 (0.066%)	417 (0.083%)	293 (0.05%)
**Exon rate (count)**	16,997 (3.749%)	18,666 (3.713%)	21,620 (3.683%)
**Intron rate (count)**	3015 (0.665%)	3400 (0.676%)	4340 (0.739%)

## Data Availability

The sequencing data generated in this study are available in the NCBI Sequence Read Archive (SRA) (BioProject accession number PRJNA1436041). The individual datasets can be accessed using SRA accession numbers SAMN56459848-SAMN56459850.

## Supplementary Materials

The following supporting information can be downloaded at: https://www.mdpi.com/article/10.3390/jof12050302/s1, Table S1: List of *Aspergillus fumigatus* strains in the study; Table S2: Primers used for PCR amplification and DNA sequencing of the *cyp51A*, *HMG1*, and the *FKS1* gene and its promoter region in *Aspergillus fumigatus*. Table S3. PCR thermocycling conditions used for amplification of the *cyp51A*, *HMG1*, and *FKS1* genes and their promoter regions in *Aspergillus fumigatus*.

## Abbreviations

The following abbreviations are used in this manuscript:

***A. fumigatus***	*Aspergillus fumigatus*
**Af293**	*Aspergillus fumigatus* reference strain Af293
**AtrR**	*A. fumigatus* transcriptional regulator
**CTAB**	Cetyltrimethylammonium bromide
**CDR1B**	Candida drug resistance protein 1B
**CLSI**	Clinical and Laboratory Standards Institute
**DNA**	Deoxyribonucleic acid
**ERG**	Ergosterol biosynthesis pathway gene
**FKS1**	1,3-β-D-glucan synthase catalytic subunit gene
**GTF**	Gene transfer format
**GFF3**	General feature format version 3
**HMG1**	3-hydroxy-3-methylglutaryl–coenzyme A reductase gene
**IA**	Invasive aspergillosis
**MEC**	Minimum effective concentration
**MIC**	Minimum inhibitory concentration
**NCCP**	National Culture Collection for Pathogens
**PVP**	Polyvinylpyrrolidone
**PCR**	Polymerase chain reaction
**RNA**	Ribonucleic acid
**SNP**	Single-nucleotide polymorphism
**TE buffer**	Tris-EDTA buffer
**TR34**	34-bp tandem repeat
**TR46**	46-bp tandem repeat
**WGS**	Whole-genome sequencing

## References

[1] WHO (2022). WHO Fungal Priority Pathogens List to Guide Research, Development and Public Health Action.

[2] Patterson T.F., Thompson G.R., Denning D.W., Fishman J.A., Hadley S., Herbrecht R., Kontoyiannis D.P., Marr K.A., Morrison V.A., Nguyen M.H. (2016). Practice guidelines for the diagnosis and management of aspergillosis: 2016 update by the Infectious Diseases Society of America. Clin. Infect. Dis..

[3] Herbrecht R., Denning D.W., Patterson T.F., Bennett J.E., Greene R.E., Oestmann J.-W., Kern W.V., Marr K.A., Ribaud P., Lortholary O. (2002). Voriconazole versus amphotericin B for primary therapy of invasive aspergillosis. N. Engl. J. Med..

[4] Dladla M., Gyzenhout M., Marias G., Ghosh S. (2024). Azole resistance in *Aspergillus fumigatus*—Comprehensive review. Arch. Microbiol..

[5] Fengler A., Bader O., Cornely O., Kurzai O., Meis J.F., Morio F., Hamprecht A. (2026). Global epidemiology of azole resistance in *Aspergillus fumigatus*. JAC Antimicrob. Resist..

[6] Howard S.J., Cerar D., Anderson M.J., Albarrag A., Fisher M.C., Pasqualotto A.C., Laverdiere M., Arendrup M.C., Perlin D.S., Denning D.W. (2009). Frequency and evolution of azole resistance in *Aspergillus fumigatus* associated with treatment failure. Emerg. Infect. Dis..

[7] Lavergne R.-A., Albassier M., Hardouin J.-B., Alvarez-Moreno C., Pagniez F., Morio F., Le Pape P., Ourliac-Garnier I. (2022). Impact of TR34/L98H, TR46/Y121F/T289A and TR53 alterations in azole-resistant *Aspergillus fumigatus* on sterol composition and modifications after in vitro exposure to itraconazole and voriconazole. Microorganisms.

[8] Lee Y.-J., Shim H.S., Cho Y.S., Sohn Y.J., Hyun J.H., Baek Y.J., Kim M.H., Kim J.H., Ahn J.Y., Jeong S.J. (2020). Identification of fungal species and detection of azole-resistance mutations in the *Aspergillus fumigatus* cyp51A gene at a South Korean hospital in Young Jung. Yonsei Med. J..

[9] Pham C.D., Reiss E., Hagen F., Meis J.F., Lockhart S.R. (2014). Passive Surveillance for Azole-Resistant *Aspergillus fumigatus*, United States, 2011–2013. Emerg. Infect. Dis..

[10] Garcia-Rubio R., Cuenca-Estrella M., Mellado E. (2017). Triazole Resistance in Aspergillus Species: An Emerging Problem. Drugs.

[11] Cubero O.F., Crespo A.N.A., Fatehi J., Bridge P.D. (1999). DNA extraction and PCR amplification method suitable for fresh, herbarium-stored, lichenized, and other fungi. Plant Syst. Evol..

[12] Bader O., Weig M., Reichard U., Lugert R., Kuhns M., Christner M., Held J., Peter S., Schumacher U., Buchheidt D. (2013). cyp51A-based mechanisms of *Aspergillus fumigatus* azole drug resistance present in clinical samples from Germany. Antimicrob. Agents Chemother..

[13] Hagiwara D., Arai T., Takahashi H., Kusuya Y., Watanabe A., Kamei K. (2018). Non-cyp51A azole-resistant *Aspergillus fumigatus* isolates with mutation in HMG-CoA reductase. Emerg. Infect. Dis..

[14] Jiménez-Ortigosa C., Moore C., Denning D.W., Perlin D.S. (2017). Emergence of echinocandin resistance due to a point mutation in the fks1 gene of *Aspergillus fumigatus* in a patient with chronic pulmonary aspergillosis. Antimicrob. Agents Chemother..

[15] Kang Y., Ma W., Li Q., Wang P., Jia W. (2025). Epidemiology, antifungal susceptibility and biological characteristics of clinical *Aspergillus fumigatus* in a tertiary hospital. Sci. Rep..

[16] Snelders E., Karawajczyk A., Schaftenaar G., Verweij P.E., Melchers W.J.G. (2010). Azole resistance profile of amino acid changes in *Aspergillus fumigatus* CYP51A based on protein homology modeling. Antimicrob. Agents Chemother..

[17] Song Y., Buil J., Rhodes J., Zoll J. (2025). Triazole-resistant *Aspergillus fumigatus* in the Netherlands between 1994 and 2022: A genomic and phenotypic study. Lancet Microbe.

[18] Novak A.R., Bradley M.E., Kiser T.H., Mueller S.W. (2020). Azole-resistant Aspergillus and echinocandin-resistant candida: What are the treatment options?. Curr. Fungal Infect. Rep..

[19] Fraczek M.G., Bromley M., Buied A., Moore C.B., Rajendran R., Rautemaa R., Ramage G., Denning D.W., Bowyer P. (2013). The cdr1B efflux transporter is associated with non-cyp51a-mediated itraconazole resistance in *Aspergillus fumigatus*. J. Antimicrob. Chemother..

[20] Paul S., Stamnes M., Thomas G.H., Liu H., Hagiwara D., Gomi K., Filler S.G., Moye-Rowley W.S. (2019). AtrR is an essential determinant of azole resistance in *Aspergillus fumigatus*. mBio.

[21] Hagiwara D., Watanabe A., Kamei K. (2016). Sensitisation of an azole-resistant *Aspergillus fumigatus* strain containing the Cyp51A-related mutation by deleting the SrbA gene. Sci. Rep..

[22] Rybak J.M., Ge W., Wiederhold N.P., Parker J.E., Kelly S.L., Rogers P.D., Fortwendel J.R. (2019). Mutations in hmg1, challenging the paradigm of clinical triazole resistance in *Aspergillus fumigatus*. mBio.

