# Supplementary material for: Molecular and Phenotypic Characterization of Multidrug-Resistant Aspergillus fumigatus Clinical Isolates in Republic of Korea
Source: J Fungi (Basel). 2026 Apr 22;12(5):302. doi: 10.3390/jof12050302 (PMC13207743; doi:10.3390/jof12050302)
Supplement: Supplementary file 1 [file jof-12-00302-s001.zip › jof-4242383-supplementary.pdf]

Table S1. List of *Aspergillus fumigatus* strains in the study.

No.	Strain (NCCP) No.	Region	Site of isolation	Year of isolation
1	21549	Gangwon-do	Ascites	2000
2	21550	Gangwon-do	Ascites	2000
3	22455	Daejeon	Ascites	N/A
4	22482	Jeonnam	Sputum	2012
5	32662	Seoul	Sputum	2015
6	32663	Seoul	Pleural	2015
7	CF3289	Gyeonggi-do	Sputum	N/A
8	CF3290	Gyeonggi-do	Bronchial	N/A
9	CF3348	Seoul	Eye	2019
10	CF3349	Seoul	Eye	2022
11	CF3350	Daegu	Pus	2019
12	MFCJUHM00002	Gyeonggi-do	Sputum	N/A
13	MFCUSHM00023	Daejeon	Urine	2018
14	MFCSSHM18224	Seoul	Sputum	2018
15	MFCDEHM00006	Jeju-do	Bronchial	2017
16	MFCUSHM00065	Gyeonggi-do	Sputum	2018

Table S2. Primers used for PCR amplification and DNA sequencing of the *cyp51A*, *HMG1*, and the *FKS1* gene and its expected product sizes in *Aspergillus fumigatus*.

Gene	Purpose	Forward Sequence (5'→3')	Reverse Sequence (5'→3')	Amplicon size (bp)
HMG1	PCR	CATTCCTGAGATTTCTCAGCATCGA	CCATGTGTATTTCGGACAGCCAGC	3,862
	Sequencing	CATTCCTGAGATTTCTCAGCATCGA	AAGAGTGATGTGGCGCTTGA	1548
	Sequencing	GCGGCGTGGATCTTGTTTTT	CTGTGATAGCGGAAGTGGCA	1033
	Sequencing	ATCGACTTGAACCCCGACAC	GGTATTGAGCTCGACCAGGG	942
	Sequencing	GCTCTCGCTGGCACTTATCT	CCATGTGTATTTCGGACAGCCAGC	991
cyp51A	PCR	TCATATGTTGCTCAGCGG	TGGATGTGTTTTTCGACCGCTT	2,076
	Sequencing	TCATATGTTGCTCAGCGG	GGGGTCGTCAATGGACTA	871
	Sequencing	CTTCTTTGCGTGCAGAGA	TCTCAGCATAATCCAGGCGC	816
	Sequencing	ACAAAAACGGCCAGCAAGTG	TGGATGTGTTTTTCGACCGCTT	729
FKS1	PCR1	GCTGAAGGATGTCGTCTGGA	CGACAATCTTGAACTTGCGA	1733
	PCR2	GAGTGGGACTGCTTCGTCAA	TACAACGGTTGACCCAGTCG	1417
	Sequencing	GCTGAAGGATGTCGTCTGGA	CCTCGCAACAGAGAAGACAG	853
	Sequencing	GGGAACGTTCTATGTCACAA	CGACAATCTTGAACTTGCGA	1009
	Sequencing	GAGTGGGACTGCTTCGTCAA	TACAACGGTTGACCCAGTCG	1417

Table S3. PCR thermocycling conditions used for amplification of the *cyp51A*, *HMG1*, and *FKS1* genes and their promoter regions in *Aspergillus fumigatus*.

Gene	Initial denaturation	Denaturation	Annealing	Extension	Final extension	Cycles
HMG1	98°C for 30 sec	98°C for 10 sec	63°C for 30 sec	72°C for 2 min	72°C for 10 min	30
cyp51A	98°C for 30 sec	98°C for 10 sec	61°C for 30 sec	72°C for 1 min	72°C for 10 min	30
FKS1	98°C for 30 sec	98°C for 10 sec	64°C for 30 sec	72°C for 1 min	72°C for 10 min	30
	98°C for 30 sec	98°C for 10 sec	65°C for 30 sec	72°C for 45 sec	72°C for 10 min	30
